# Monitoring of micturition and bladder volumes can replace routine indwelling urinary catheters in children receiving intravenous opioids: a prospective cohort study

**DOI:** 10.1007/s00431-020-03703-7

**Published:** 2020-06-11

**Authors:** Anita C. de Jong, Jolanda M. Maaskant, Luitzen A. Groen, Job B. M. van Woensel

**Affiliations:** 1 Pediatric Intensive Care Unit, Emma Children’s Hospital, Amsterdam UMC, University of Amsterdam, PO Box 22660, 1100 DD Amsterdam, the Netherlands; 2 Department of Clinical Epidemiology, Biostatistics and Bioinformatics, Amsterdam UMC, University of Amsterdam, Amsterdam, the Netherlands; 3 Department of Pediatric Urology, Emma Children’s Hospital, Amsterdam UMC, University of Amsterdam, Amsterdam, the Netherlands

**Keywords:** Urinary retention, Urinary catheter, Analgesics, Opioid [pharmacological action], Morphine, Child

## Abstract

In this prospective observational study, the incidence, risk factors and the time to event of urinary retention in children receiving intravenous opioids were evaluated. Urinary retention was confirmed by ultrasound following the inability to void for 8 h or earlier in patients experiencing discomfort. In total, 207 opioid episodes were evaluated, of which 199 (96.1%) concerned morphine, in 187 children admitted to the pediatric ward or pediatric intensive care unit. The median age was 7.6 years (IQR 0.9–13.8), and 123 (59.4%) were male. The incidence of urinary retention was 31/207 (15.0%) opioid episodes, in which 14/32 (43.8%) patients received continuous sedation for mechanical ventilation and 17/175 (9.7%) received no sedation. Multivariable logistic regression analysis showed a significant association with continuous sedation (OR 6.8, 95% CI 2.7–17.4, *p* 0.001) and highest daily fluid intake (OR 0.8 per 10% deviation of normal intake, 95% CI 0.7–0.9, *p* 0.01). Opioid dosage, age and gender were not significantly associated. Most events (28/31, 90.3%) occurred within 24 h.

*Conclusion*: The incidence of urinary retention in children receiving intravenous opioids is low, indicating that placement of urinary catheters is not routinely necessary in these patients. However, micturition and bladder volumes must be monitored, especially in sedated children and during the first 24 h of opioid administration.

**Table:** 

**What is Known:** • *Great variation exists in the routine placement of urinary catheters in children receiving IV opioids.*	
**What is New:** **•** *Confirmed by ultrasound, the incidence of urinary retention in children receiving intravenous opioids in this study was 15%, indicating that placement of urinary catheters is not routinely necessary in these patients.* **•** *Children receiving continuous sedation for invasive mechanical ventilation showed a sevenfold greater risk of developing urinary retention than non-sedated patients.*

**Electronic supplementary material:**

The online version of this article (10.1007/s00431-020-03703-7) contains supplementary material, which is available to authorized users.

## Introduction

Opioids are widely used for the management of pain and for sedation in children. However, opioids can cause acute urinary retention [1]. Urinary retention may cause pain or discomfort, but this is often masked by the opioid [2]. Although pharmacological management of opioid-related urinary retention is described in some studies [3], it often requires bladder catheterization to prevent over-distension which may result in long-term bladder dysfunction [2]. Urinary retention may be prevented by placement of an indwelling urinary catheter. However, this is in itself associated with discomfort, impaired mobilization and catheter-associated urinary tract infections (CAUTI). The rate of CAUTI in children varies strongly and may be up to 32 per 1000 catheter days [4].

The reported incidence of urinary retention in children receiving intravenous (IV) opioids varies widely from 1 to 64% [5–12]. This wide range is most likely explained by differences in patient characteristics and criteria for defining urinary retention. Yet the incidence has only been studied in primarily small cohorts of postoperative patients above 3 months old in non-critical care settings. In addition, risk factors in children have been scarcely studied. Consequently, great variation exists in the routine placement of indwelling urinary catheters in children receiving IV opioids [11]. The results of two recent studies indicated that urinary catheters are not routinely necessary in children receiving patient-controlled analgesia (PCA) following appendicitis [9, 11]. However, none of the previous studies included children of all ages and children receiving continuous sedation for the facilitation of invasive mechanical ventilation.

Strategies to reduce inappropriate use of indwelling urinary catheters including strict indications for placement and removal contribute to reducing the risk of catheter-related complications and their associated costs. Therefore, the aim of this study is to evaluate the incidence of urinary retention in children receiving IV opioids, in order to increase our insight into the necessity for placement of a urinary catheter during opioid treatment. Secondary objectives are to identify independent risk factors for urinary retention and to establish the time to event. We hypothesize that children receiving IV opioids do not routinely require an indwelling urinary catheter.

## Materials and methods

### Study design and setting

This prospective observational study was performed at a tertiary children’s hospital from January 2018 to November 2018. The study was approved by the local institutional review board (FWA00025191).

Patients were selected based on the following inclusion criteria: age 0–18 years old, treatment with IV opioids continuously and/or via PCA for a minimum of 4 h. Both patients admitted to the pediatric wards and pediatric intensive care unit (PICU) could be included. Each patient could be included once per hospital admission. If the initial indication for a urinary catheter was no longer present, patients who still received IV opioids were also included following removal of the catheter. Patients were excluded following occurrence of intra-operative urinary retention, by known urological pathology; if conduction of bladder ultrasound was not possible; epidural, spinal or caudal anaesthesia in the preceding 24 h; treatment with paralytic agents; neuro-muscular diseases; prematurity (< 37 weeks of gestation) or admission to the neonatal intensive care unit. In line with Dutch legislation, written informed consent was obtained from parents or legal guardians of patients under 16 years of age, and in patients of 12 years or older also from the patients themselves.

Acute urinary retention was defined as the inability to void for the duration of 8 h, or earlier in cases experiencing abdominal discomfort, in combination with a larger than expected for age bladder volume estimated by ultrasound scan (< 1 year: [kg × 7 mL], ≥ 1 year: [(age in years + 2) × 30 mL]) or above 400 mL [13, 14]. Bladder volumes were estimated twice by an automated bladder ultrasound device (Verathon Bladderscan Prime® [15]), further referred to as ‘Bladderscan®.’ In addition, due to the reported unreliable Bladderscan® measurement in children below 25 kg or under 7 years of age [16, 17], in patients under 10 years of age, a conventional bladder ultrasound scan (M-Turbo, Fujifilm SonoSite Inc.® [18]) was made. Bladder volumes (millilitres) measured with a conventional bladder ultrasound were calculated as depth × height × width × 0.68 (centimetres) [19]. The greatest estimated volume measured with either conventional bladder ultrasound or Bladderscan® was used to evaluate the bladder volume.

Toilet-trained children with a bladder volume larger than expected were first encouraged to void spontaneously for 30 min before diagnosing urinary retention. Depending on their mobility, a toilet, urinal, bedpan or diaper was used in a private room, when necessary assisted by a nurse or parent/legal guardian, according to the child’s preference.

Data on urinary retention-related pain or discomfort were collected from nursing patient reports. In patients admitted to the PICU, discomfort was evaluated by the nurses using the standardised COMFORT behavioural scale. In children admitted to the general pediatric wards, discomfort was evaluated by the nurses using the numerical rating scale, visual analogue scale or FLACC behavioural scale, depending on the patients’ level of communication and awareness.

Follow-up of patients was carried out until 12 h after the cessation of IV opioids, with a maximum of 7 days after initiating the IV opioids. In cases in which urinary retention occurred, an indwelling urinary catheter was placed for the remaining duration of IV opioid treatment. Additionally, the initial catheterized bladder volume was recorded.

The variables that were considered as potential risk factors for developing urinary retention in children receiving IV opioids are shown in Table 2. These variables were selected based on the available literature and biological plausibility.

### Statistical analysis

Data was analysed using R version 3.6.1 [20]. Continuous data with a normal distribution was reported as mean and standard deviation (SD), otherwise as median and interquartile range (IQR).

The incidence of urinary retention was calculated for episodes of IV opioids of the overall cohort, and for subgroups with evaluated risk factors. Univariable and multivariable binomial logistic regression analyses were performed to study the association between potential risk factors and the occurrence of urinary retention. A *p* value of < 0.05 was considered statistically significant. Log-linearity of continuous variables was evaluated via visual inspection, categorization and testing of non-linear transformations. In cases of non-log-linearity, categorization of variables was conducted. Variables with a *p* value < 0.1 in the univariable analysis were included into a multivariable model. Multi-collinearity of variables was evaluated by a variance inflation factor below five. A maximum ratio of ten events per variable was applied in the model. If more variables were eligible, decision for inclusion was based on the *p* value, the variance inflating factor and clinical relevance. Variable selection was performed via stepwise backward selection. Risk factors only present in subgroups were only univariable analysed.

Opioid dose included continuously administered IV opioids, IV boluses and PCA and was analysed per 10 mcg/kg/h. If opioids other than IV morphine were administered, the equivalent dose was calculated [21]. If patients switched between morphine and another type of IV opioid, the type of opioid was evaluated as morphine. Peak opioid dose was analysed over four consecutive hours. Mean opioid dose was analysed until the occurrence of urinary retention. In patients in whom there were no signs of urinary retention, the opioid dose was analysed until the median time to the event of the overall cohort. Highest daily fluid intake was obtained from the day during the study period with the highest fluid intake and was analysed per 10% deviation of common practice of normal intake for age and bodyweight [22].

The time to event was analysed by cumulative incidence from initiating the IV opioids or following removal of the initial urinary catheter, until the occurrence of urinary retention or until 12 h after the cessation of IV opioids, with a maximum follow-up of 7 days.

Handling of missing data is provided in the Online Resource.

## Results

### Participants

A total of 231 patients, receiving 252 IV opioid episodes, were eligible for participation. A total of 187 patients went on to be included, of whom 207 opioid episodes were evaluated. Of these 187 patients, 170 (90.9%) were included once, 16 (8.6%) were included twice and one (0.5%) was included five times. Forty-five opioid episodes were excluded for reasons of failure to include the patient within 8 h after initiation of IV opioids (*n* = 27), declined informed consent (*n* = 9), social circumstances (*n* = 6) and language barrier (*n* = 3). The patient characteristics are summarized in Table 1.

**Table 1:** Patient characteristics

	*n* = 207 opioid episodes, related to 187 individual patients
Age (years), median (IQR)	7.6 (0.9–13.8)
Bodyweight (kg), median (IQR)	24.0 (9.3–54.4)
Male gender, %	59.4
Ethnicity, %	
Caucasian	74.4
Black	14.0
North African/Arabic	3.4
Other/mixed/unable to obtain	8.2
Reason for admission, %	
Surgical	51.7
Respiratory	11.1
Circulatory/cardio-surgery	4.8
Neurology/neuro-surgery	7.7
Hematology	10.6
Oncology	8.2
Trauma	3.4
Other	2.5
Localization of surgery, %^a^	
Head	8.0
Facial/mouth	13.9
Ear, nose, throat	2.1
Thoracic	19.6
Abdominal	32.8
Orthopedic	19.0
Other	4.6
Anaesthesia, %^b^	58.9
Hospital length of stay (days), median (IQR)	6.0 (4.0–11.0)
PICU admission, %	31.4
PRISM III score, median (IQR)^c^	2.0 (0.0–5.0)
Continuous sedation, for invasive mechanical ventilation, %	15.4
28-day mortality, %	1.0
Type of IV opioid, %	
Morphine, or switch between morphine and other	96.1
Buprenorphine only	2.9
Fentanyl only	1.0
Administration type of IV opioid, %	
Basal rate only	27.5
PCA only	21.7
Basal rate and PCA/boluses	50.8
IV opioid dose (morphine equivalent, mcg/kg/h), median (IQR)^d, e^	14.0 (9.0–21.4)
Duration of IV opioids (h), median (IQR)^d^	43.2 (20.6–71.2)
Co-medication associated with urinary retention, %	
Co-medication, overall	74.9
Anticholinergic drugs	12.1
Benzodiazepines	6.8
Non-steroid anti-inflammatory drugs	53.6
Alpha-adrenoreceptor agonists (continuously)	17.4
Other (theophylline, vincristine, baclofen, estrogens)	3.9
Removal of indwelling urinary catheter^f^	11.1

*IQR* interquartile range, *IV* intravenous, *PCA* patient-controlled analgesia, *PICU* pediatric intensive care unit, *PRISM score* pediatric risk of mortality score

^a^Seven days preceding or during the study period (*n* = 137)

^b^Twenty-four hours preceding or during the study period

^c^In PICU patients (*n* = 65)

^d^Cases: until the occurrence of individual urinary retention, non-cases: until the median time to event of the overall cohort (9.0 h)

^e^Excluding intra-operative opioids

^f^Patients were included following removal of the catheter

The median observation time was 43.2 h (IQR 20.6–71.2). Follow-up was continued until cessation of IV opioids in 192/207 (92.8%) episodes. In 7/207 (3.4%) episodes, follow-up was terminated prematurely after a median of 38.5 h (IQR 18.0–60.5) due to hospital discharge or medical indication for placement of a urinary catheter. The observed period of these episodes was included in our analysis. Follow-up was discontinued in 8/207 (3.9%) episodes in which patients received IV opioids for more than 7 days without occurrence of urinary retention.

### Incidence of urinary retention

In total, spontaneous voiding occurred at least every 8 h in 153/207 (73.9%) IV opioid episodes. In 52 episodes, a total of 59 ultrasound scans were performed, in which the bladder volume was larger than expected for age in 35. In four episodes, patients voided spontaneously within 30 min following performance of the abdominal ultrasound. Therefore, the overall incidence of urinary retention in episodes of children receiving IV opioids was 31/207 (15.0%), in which 14/32 (43.8%) episodes the patient received continuous sedation for the facilitation of invasive mechanical ventilation and in 17/175 (9.7%) episodes patients did not receive continuous sedation. Two patients, both of whom were included twice, developed urinary retention only during one opioid episode. Therefore, the overall number needed to treat was one case requiring placement of a urinary catheter per seven episodes of IV opioids. In patients receiving continuous sedation for the facilitation of invasive mechanical ventilation, the number needed to treat was one catheter per two opioid episodes and in patients who did not receive continuous sedation one catheter per 11 opioid episodes.

In patients who developed urinary retention, the median estimated bladder volume was 138% (IQR 111–162) of expected maximum bladder capacity for age. Of these cases, 14/31 (45.2%) experienced abdominal pain or noticeable discomfort.

### Risk factors

The univariable analyses of potential risk factors are presented in Table 2, and the multivariable model is presented in Table 3. The variables ‘reason for admission’ and ‘PICU admission,’ that were both statistically significant in univariable analysis, were not included in the multivariable model for the following reasons. Patients with the admission criterion ‘respiratory’ were all PICU patients requiring invasive mechanical ventilation. Therefore, in the multivariable model, we only included ‘receiving continuous sedation for the facilitation of invasive mechanical ventilation.’ Since 32/65 (49.2%) of all participating PICU patients were mechanically ventilated and the criteria for PICU admission vary amongst centres, we excluded PICU admission in the multivariable model. For reasons of clinical relevance, we included mean opioid dose in the multivariable model despite its non-significance in the univariable analysis.

**Table 2:** Univariable analyses of risk factors associated with acute urinary retention in children receiving IV opioids

Variables	Beta	OR	95% CI	*p* value
Age, years	− 0.0	0.8	0.9–1.0	0.47
Bodyweight, kg	− 0.0	1.0	1.0–1.01	0.40
Gender—male	0.1	1.1	0.5–2.5	0.82
Ethnicity				
Caucasian	1.0	–	–	–
North African/Arabic	− 0.6	0.9	0.0–5.6	0.89
Black	− 0.2	0.9	0.2–2.5	0.74
Mixed/other/unable to obtain	− 1.1	0.3	0.0–1.8	0.28
Reason for admission				
Surgery	1.0	–	–	–
Respiratory	1.2	3.5	1.1–10.0	0.02*
Circulatory/cardio-surgery	0.7	2.0	0.3–9.1	0.42
Neurology/neurosurgery	1.3	3.6	1.0–11.9	0.04*
Haematology	− 0.2	0.8	0.2–3.2	0.77
Oncology	− 15.5	0.0	NA	0.99
Trauma	1.6	3.2	0.4–16.6	0.56
Other	0.7	2.0	0.1–14.8	0.55
Hospital length of stay, days	− 0.0	1.0	1.0–1.0	0.62
PICU admission	1.5	4.5	2.0–10.2	< 0.001*
PRISM III score^a^	− 0.0	0.9	0.8–1.1	0.53
Continuous sedation, for mechanical ventilation	2.0	7.2	3.1–17.2	< 0.001*
Opioid type IV				
Morphine, or switch between morphine and other	1.0	–	–	–
Buprenorphine only	− 15.8	0.0	NA	0.99
Fentanyl only	− 15.8	0.0	NA	1.00
Administration type of IV opioids				
Basal rate only	1.0	–	–	–
PCA only	− 0.1	0.9	0.3–2.9	0.92
Basal rate and PCA/bolus	0.2	1.2	0.5–3.1	0.72
Opioid dose				
Mean morphine equivalent dose, 10 mcg/kg/h^b^	0.2	1.2	1.0–1.4	0.11
Mean morphine equivalent dose, 10 mcg/kg/h^c^	0.0	1.0	0.9–1.1	0.78
Peak morphine equivalent dose, 10 mcg/kg/h^b,d,e^	0.1	1.1	1.0–1.1	0.33
Peak morphine equivalent dose, 10 mcg/kg/h^c,d,e^	0.0	1.0	1.0–1.1	0.67
Anaesthesia^f^	− 0.2	0.8	0.4–1.8	0.62
Intra-operative morphine equivalent dose, 100 mcg/kg/h	0.3	1.3	1.0–1.6	0.03*
Duration of anaesthesia, h	− 0.2	0.8	0.5–1.7	0.35
Intra-operative fluid replacement, 10 ml/kg	0.3	1.4	1.0–1.9	0.04*
Localization of surgery^g^				
No surgery	1.0	–	–	–
Head	0.6	1.8	0.4–7.4	0.43
Mouth, facial or ear, nose, throat	17.0	0.0	NA	0.99
Thoracic	0.3	1.4	0.4–4.0	0.57
Abdominal	− 0.5	0.6	0.2–1.8	0.37
Orthopaedic	− 0.1	0.9	0.3–2.8	0.84
Other	− 0.0	1.0	0.1–6.8	0.98
Neurological illness	0.3	1.4	0.5–3.5	0.52
Fluid intake				
Highest daily fluid intake^h^	− 0.3	0.8	0.7–0.9	0.002*
Mean daily fluid intake^i^				
80–120%	1.0	−	–	–
< 80%	0.7	2.0	0.7–5.2	0.16
> 120%	− 1.2	0.3	0.1–1.1	0.11
Removal of indwelling urinary catheter^j^	0.2	1.2	0.3–3.6	0.73
Toilet trained	− 0.2	0.8	0.4–1.1	0.55
Voiding-related mobility^k^				
Bedrest	1.0	−	–	–
Bedside	0.1	1.1	0.3–3.5	0.89
Mobile for toilet	− 1.5	0.2	0.0–1.3	0.17
Co-medication associated with urinary retention				
Co-medication, overall	0.9	2.4	0.9–8.5	0.10
Anticholinergic drugs	0.4	1.5	0.5–4.1	0.41
Benzodiazepines	− 0.9	0.4	0.0–2.2	0.43
Non-steroid anti-inflammatory drugs	− 0.3	0.8	0.4–1.7	0.53
Alpha-adrenoreceptor agonists (continuously)	0.2	1.2	0.4–2.9	0.76
Other (theophylline, vincristine, baclofen, estrogens)	− 0.2	0.8	0.0–4.8	0.84

*CI* confidence interval, *IQR* interquartile range, *IV* intravenous, *OR* odds ratio, *PICU* pediatric intensive care unit, *PCA* patient-controlled analgesia, *PRISM score* pediatric risk of mortality score

*p value < 0.05

^a^Of PICU patients (*n* = 65)

^b^Excluding intra-operative opioids

^c^Including intra-operative opioids

^d^Peak of 4-h mean dose

^e^Cases: until the occurrence of urinary retention; non-cases: until the median time to event of the overall cohort (9.0 h)

^f^Twenty-four hours preceding or during the study period

^g^Seven days preceding or during the study period

^h^Per 10% deviation of normal intake for age and bodyweight, obtained per patient from the day during the study period with the highest fluid intake

^i^Percentage of deviation of normal intake for age and bodyweight, obtained from the mean daily fluid intake

^j^Patients were included following removal of the catheter

^k^If toilet trained (*n* = 117)

**Table 3:** Multivariable analysis of risk factors associated with acute urinary retention in children receiving IV opioids

Variables	Beta	Adjusted OR	95% CI	*p* value
Continuous sedation, for mechanical ventilation	1.9	6.8	2.7–17.4	< 0.001*
Highest daily fluid intake^a^	− 0.2	0.8	0.7–0.9	0.01*
Mean morphine equivalent dose, 10 mcg/kg/h^b^	0.2	1.3	1.0–1.6	0.07

*CI* confidence interval, *IV* intravenous, *OR* odds ratio

*p value < 0.05

^a^Per 10% deviation of normal intake for age and bodyweight, obtained per patient from the day during the study period with the highest fluid intake

^b^Excluding intra-operative opioids

Following stepwise backward logistic regression analysis, only continuous sedation for the facilitation of mechanical ventilation (adjusted OR = 6.8, 95% CI 2.7–17.4, *p* < 0.001) and highest daily fluid intake (adjusted OR = 0.8 per 10% deviation of the normal intake, 95% CI 0.7–0.9, *p* = 0.01) remained as statistically significant factors associated with the subsequent acquirement of urinary retention. The variance inflating factors were below 1.1, indicating no multi-collinearity amongst variables.

### Time to event

**Fig. 1:**
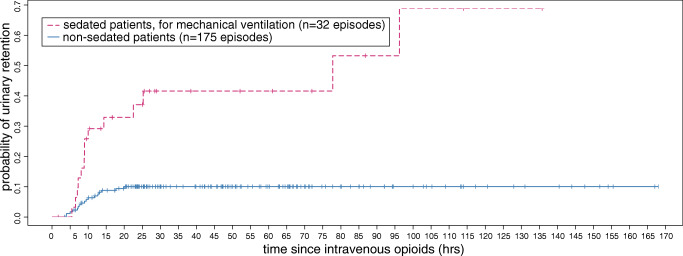
Cumulative incidence of urinary retention in children receiving IV opioids

The overall median time to event was 9.0 h (IQR 7.1–13.3), with 9.0 h (IQR 7.5–20.5) in episodes of patients receiving continuous sedation for the facilitation of invasive mechanical ventilation and 8.5 h (IQR 6.7–12.8) in episodes of patients who did not receive continuous sedation (Fig. 1). In 28/31 (90.3%) cases, the urinary retention occurred within 24 h following IV opioid administration or removal of the urinary catheter. Two patients receiving continuous sedation for the facilitation of invasive mechanical ventilation developed urinary retention 3 days after the initiation of IV opioids, both in the absence of any opioid dose adjustments but initiation of benzodiazepines in one case. Two teenage cases refused placement of a urinary catheter and eventually voided spontaneously overnight.

### Missing data

We could not perform a reliability test, because in patients who developed urinary retention, the time between performing the bladder ultrasound scan and the initial bladder volumes measured following catheterization varied.

The missing data are presented in the Online Resource.

## Discussion

In children receiving IV opioids, we found that acute urinary retention, as confirmed by bladder ultrasound scan, occurred in 15.0% of episodes. Previous studies describe an incidence ranging from 1 to 64% [5–12]. However, ultrasound confirmation of bladder volumes was performed in only two of these previous studies [8, 11]. Alfheim et al. reported an incidence of 64% following cleft palate surgery, which they hypothesized to be related to high dosages of post-operative morphine, but this was not analysed [8]. In their study, despite the fact that this technique is known to be inaccurate in children below 25 kg or under 7 years old [16, 17], Bladderscan® was used even though all children were aged less than 20 months. Sobrino et al. found an incidence of 4%, confirmed by ultrasound scan after clinical symptoms in children receiving PCA following appendicitis [11]. Since over half of our cases experienced no evident abdominal pain or discomfort, the single criterion of clinical symptoms used in the latter study may explain the higher incidence of urinary retention in our study population.

Since none of the previous studies included children receiving continuous sedation for the facilitation of invasive mechanical ventilation, we cannot compare the incidence of 43.8% we found in this subgroup. Our results suggest that these children have a sevenfold greater risk of developing urinary retention than non-sedated patients. The large number of critically ill patients included in this study could in part explain these results. It is known that critically ill children may have reduced physiological clearance of opioids due to impaired hepatic and/or renal function [23, 24]. In addition, encouragement of spontaneous voiding may not always be possible in sedated patients. In contrast to the study of Verhamme et al. [1], we did not observe a significant risk of reported co-medication, although invasively mechanically ventilated patients often receive poly-pharmacology.

Unexpectedly, we found a negative association of highest daily fluid intake and urinary retention. Although speculative, a high daily fluid intake might be positively associated with renal opioid clearance or dilution of active opioid metabolites. Alternatively, a low frequency of voiding in children with low daily fluid intake might have resulted in observing a full bladder, especially in children in whom encouragement of spontaneous voiding was not possible.

In contrast to the general assumption that a dose-response relationship exists between opioids and occurrence of urinary retention, in line with previous pediatric and adult studies, we failed to demonstrate this relationship [9, 12, 25]. Potentially, there could be a certain threshold above which the dose of opioid may not give any further increased risk of urinary retention. In line with the reported studies in children over 5 years old [9, 12], we neither could demonstrate age as an independent risk factor. As morphine clearance is positively correlated with gestational age [26], one could speculate that neonates may be at greater risk of developing urinary retention. Since we only included 11 neonates, we were unable to confirm this hypothesis. Controversy exists regarding the impact of gender on the development of urinary retention. In line with some other studies [9, 12], we too were unable to demonstrate an association with gender. In contrast, Gatti et al. reported a threefold increase in drug-related urinary retention in boys compared to girls [27].

To our knowledge, the time to development of urinary retention following the commencement of IV opioids has not been previously studied, whereas we believe that this information may be of great clinical relevance for the monitoring of spontaneous micturition. We identified a median time to event of 9 h after initiating the IV opioids or removal of the initial urinary catheter and in 90% of the cases this occurred within the first 24 h. If adverse effects are more likely to be related to morphine itself rather than the slowly accumulating effect of its more potent active metabolite morphine-6-glucuronide [28], this might support our observation that urinary retention mainly occurs during the first 24 h of opioid administration.

The major strengths of our study are the size of the study population and the clear definition of urinary retention including confirmatory ultrasound scan. Nevertheless, the following limitations should be considered when interpreting our results. Firstly, due to the lack of a uniform definition, and as urinary retention often remains asymptomatic [2, 29], the observed incidence in our study might be an overestimate when compared to studies using the definition ‘the requirement for catheterization’ [5, 9, 10, 12]. Secondly, since the incidence in previous studies varies widely, we were unable to make a reliable power calculation. Given the low observed incidence of urinary retention in our study, we were limited to a multivariable analysis of three variables. Thirdly, identified risk factors may have been confounded by pre-opioid bladder volume [25], which was not analysed for reasons of feasibility. Finally, the identified time to event might have been influenced by our definition of urinary retention following an absence of spontaneous voiding over an 8-h interval. One unresolved question which remains is whether urinary retention in children occurs as a single or repeated event.

In conclusion, a low incidence of urinary retention in children receiving IV opioids was observed, indicating that indwelling urinary catheters are not routinely necessary in these patients. However, micturition and bladder volumes must be well monitored, especially in mechanically ventilated children receiving continuous sedation, who appeared to have an increased risk of developing urinary retention. This monitoring is of the greatest importance during the first 24 h following IV opioid administration or removal of the urinary catheter. Development of a uniform definition of urinary retention in children is necessary in order to conduct further research into risk factors.

## Electronic supplementary material

ESM 1 (PDF 411 kb)

## Abbreviations

CAUTI: Catheter-associated urinary tract infection
CI: Confidence interval
IQR: Interquartile range
IV: Intravenous
OR: Odds ratio
PCA: Patient-controlled analgesia
PICU: Pediatric intensive care unit
