# Supplementary material for: Monitoring of micturition and bladder volumes can replace routine indwelling urinary catheters in children receiving intravenous opioids: a prospective cohort study
Source: Eur J Pediatr. 2020 Jun 11;180(1):47–56. doi: 10.1007/s00431-020-03703-7 (PMC7782390; doi:10.1007/s00431-020-03703-7)
Supplement: Supplementary file 1 — (PDF 411 kb) [file 431_2020_3703_MOESM1_ESM.pdf]

Online Resource: Missing data

Article Monitoring of micturition and bladder volumes can replace routine indwelling urinary catheters in children receiving intravenous opioids.
A prospective cohort study

Journal European Journal of Pediatrics
Authors Anita C. de Jong[1], Jolanda M. Maaskant, Luitzen A. Groen, Job B.M. van Woensel
[1)] *Pediatric Intensive Care Unit, Emma Children's Hospital, Amsterdam UMC, University of Amsterdam, Amsterdam, the Netherlands,* A.C.dejong@amsterdamumc.nl

Missing data: methods

Missing PCA values were imported as mean from individual adjacent hours of PCA usage. If daily fluid intake was not well documented it was assumed to be normal (100%) for age and bodyweight. If an ultrasound scan was not performed within the given time-frame, patients were classified as non-cases, providing they spontaneously voided within 12 hours since initiating the IV opioids or removal of the initial urinary catheter. A worst-case analysis was performed in patients evaluated as non-case without confirmatory ultrasound scan. A sensitivity analysis was performed of missing daily fluid intake, by comparing imputation of the first and third quartile of administered daily fluid intake.

Missing data: results

Opioid dose in PCA usage was missing in 276/10802 (2.6%) of observed hours. Daily fluid intake was incomplete in 189/686 (27.6%) patient-days. The IQR of administered maximum daily fluid intake was 100-140% of normal intake for age and bodyweight. If missing daily fluid intake was imputed as 140% of normal daily fluid intake, its risk remained statistically significant in both uni- and multivariable analysis.

Of the performed ultrasound scans, 14/59 (27.7%) were performed using only a Bladderscan® due to absence of staff trained in performing a conventional bladder ultrasound scan. Four of these patients were under three years of age. In two patients an ultrasound scan was not performed at all. They were classified as non-cases, since they spontaneously voided within 12 hours since initiating the IV opioids. If both patients would have been classified as case, the overall incidence would have been 15.9%, with 43.8% in episodes of patients receiving continuous sedation for the facilitation of invasive mechanical ventilation, and 10.9% non-sedated patients. Identified significant risk factors and time to event would have remained equal.
